# HEPATIC STELLATE CELLS ARE A MAJOR COLLAGEN PRODUCING CELLS IN THE LIVER AND DRIVE LOCAL LIVER FIBROSIS

**DOI:** 10.1093/geroni/igae098.2254

**Published:** 2024-12-31

**Authors:** Victory Iluobe, Shangang Zhao

**Affiliations:** Howard University, windsor mill, Maryland, United States; university of texas barshop institution, university of texas barshop institution, Texas, United States

## Abstract

Hepatic stellate cells (HSCs) are the major collagen-producing cells in the liver and key drivers of local fibrosis. Adiponectin, is also expressed in quiescent HSCs and plays a role in the progression of liver fibrosis. To assess adiponectin’s effects on fibrosis progression mice line models were crossed using these models liver fibrosis progression either progressed or reduced. We moreover show that adiponectin expression in HSCs correlates positively with that of PPARγ, suggesting a mechanistic involvement of this transcription factor. The involvement of this transcription factor has a marked influence on the progression of local fibrosis.

